# Childhood Immunization and COVID-19: An Early Narrative Review

**DOI:** 10.3389/fpubh.2020.587007

**Published:** 2020-10-28

**Authors:** Bojana Beric-Stojsic, Julie Kalabalik-Hoganson, Denise Rizzolo, Sanjoy Roy

**Affiliations:** MPH Program, School of Pharmacy and Health Sciences, Fairleigh Dickinson University, Madison, NJ, United States

**Keywords:** children, vaccines, COVID-19, SARS-CoV-2, immunization

## Abstract

The COVID-19 pandemic has evolved into arguably the largest global public health crisis in recent history—especially in the absence of a safe and effective vaccine or an effective anti-viral treatment. As reported, the virus seems to less commonly infect children and causing less severe symptoms among infected children. This narrative review provides an inclusive view of scientific hypotheses, logical derivation, and early analyses that substantiate or refute such conjectures. At the completion of a relatively less restrictive search of this evolving topic, 13 articles—all published in 2020, were included in this early narrative review. Directional themes arising from the identified literature imply the potential relationship between childhood vaccination and COVID-19—either based on the potential genomic and immunological protective effects of heterologous immunity, or based on observational associations of cross-immunity among vaccines and other prior endemic diseases. Our review suggests that immune response to the SARS-CoV-2 virus in children is different than in adults, resulting in differences in the levels of severity of symptoms and outcomes of the disease in different age groups. Further clinical investigations are warranted of at least three childhood vaccines: BCG, MMR, and HEP-A for their potential protective role against the SARS-CoV-2 virus.

## Introduction

The novel Severe Acute Respiratory Syndrome Coronavirus 2 (SARS-CoV-2) has infected close to 9.5 million people and has claimed nearly 762,000 lives globally, as of August 16, 2020 (1). This pandemic has evolved into arguably the largest global public health crisis in recent history—especially in the absence of a safe and effective vaccine or an effective anti-viral treatment. This virus has demonstrated a high attack rate, a broad gamut of identifiable symptoms, and viability among a potentially massive number of infected silent carriers.

Unlike many infectious diseases, such as endemic malaria and common flu where children are known to have the highest mortality rates and to drive transmission in households and communities—it appears as it could be that SARS-CoV-2 just does not translate into severe disease as frequently in children, specifically for young children, below 10 years of age. Moreover, infected children suffer milder symptoms of COVID-19, with much lower case-fatality rates (CFR), and recover quickly from the infection (2–7). In an initial assessment from Wuhan, China, among 50 children identified with COVID-19, the severity varied between asymptomatic and mild in 96% of the patients (8). While diagnostic findings were similar to those of adults, fewer children developed severe pneumonia. Neonates, on the other hand, have developed symptomatic and more severe COVID-19 (2, 7).

Data obtained from the Chinese Center for Disease Control and Prevention as of February 17, Spanish Ministry of Health as of March 24, Korea's Centers for Disease Control and Prevention as of March 24, and the Italian National Institute of Health as of March 17—suggests that the CFR for COVID-19 for children are disproportionately lower compared to any other age group (9). The CFR was 0% for all four countries for the age group “0–9 years.” Singh et al., suggest that milder symptomatology implies potential immunologic protective factors in children and the direction for a design of interventions for all age groups (2). While it is likely that early publication of reports from countries with generally more equipped healthcare systems may not be fully indicative of the long-term overall potential impact in less developed nations—current observations do not suggest such trends yet.

Propositions for such lower observed rate of fatality and symptomatic illness have included the potential protective effect of global active viral immunization of children from birth till 6 years of age (10). It is suggested that childhood vaccines for mumps, rubella, poliomyelitis, Hepatitis B, and varicella may impart transient immunity against SARS-CoV-2 that protects their lung cells from contracting COVID-19 (10). Subsequently, aging, immunosuppression, and co-morbid states reduce the adaptability of the immune system (5).

The rates of heterologous immunity have been studied in some of the common childhood vaccinations including measles and Bacillus Calmette-Guerin (BCG) vaccines. African American girls who received the measles vaccine demonstrated 47% reduced mortality from other diseases. Similarly, the BCG vaccine has demonstrated a 25% reduction in mortality to other diseases (11). Previous research has supported that live vaccines have increased resistance to other vaccine unrelated diseases. Thus, they have specific effects by preventing the targeted disease but also non-specific effects on non-targeted infections as well. It is theorized that vaccines boost immune responses, offering additional resistance to viruses other than the ones they are intended to prevent (12). It should also be noted that research only suggests there is a correlation between vaccines and non-specific responses, not causation.

Such hypothesis of cross-immunogenicity of existing childhood vaccines with the novel coronavirus, if proven true, could have far reaching implications for public health immunization policies across the globe. However, no broad assessment of this topic has thus far been undertaken to the best of our knowledge at the time of this writing.

In this narrative review, we provide an inclusive view of scientific hypotheses, logical derivation, and early analyses that substantiate or refute such conjectures. The goal of this study is not to establish a comprehensive, systematic understanding of the link between childhood vaccination and COVID-19 outcomes. Instead we attempt to offer a robust starting point to facilitate further development of relevant hypotheses and designing of studies to test this promising public health opportunity.

## Methods

Given the early stage in the evolving literature on this topic, our attempt at a systematic search of health sciences databases such as PubMed using keywords and search strategies such as: “coronavirus OR COVID-19 OR nCoV OR SARS-CoV-2) AND (child OR children OR childhood OR pediatric OR infant OR babies OR baby OR neonates) AND (immunization OR vaccination OR vaccine)” limited to English language articles published in between June 2019 and April 2020 did not yield sufficiently relevant publications. While there may have been articles that were published in other languages during the review period, those were not included unless an English translation was available. Given the importance of this topic, we believe that such non-English articles, if any, will be included in future assessments as there is broader presence of the data in global literature.

As such, given the broader base of sources accessed by its search function, we performed a plain language search using the same keywords listed above on Google Scholar, which includes journal and conference papers, theses and dissertations, academic books, pre-prints, abstracts, technical reports, and other scholarly literature from all broad areas of research (13). All types of study and countries of origin were eligible for inclusion. In addition, any relevant articles that were identified during and outside the formal search process were also included if their content were relevant to our study. Four reviewers extracted relevant data into a cloud-based spreadsheet. We recorded the country of origin, study design, type of data, results, and conclusions. As this was intended to be a rapid review, each article was reviewed by one reviewer.

## Results

At the completion of the quick search and identification process, 14 out of 30 identified articles were included in this early narrative review (Table 1). Included papers were all published in 2020 following the early release of data on COVID-19, presenting hypotheses about the potential relationship between childhood vaccination and COVID-19—either based on the protective effects of heterologous immunity, or based on observational associations of cross-immunity among vaccines and other prior endemic diseases.

**Table 1:** Summary of studies included in the narrative review.

**Theme**	**References#**	**Authors**	**Country of origin of study**	**Type of study**
Immunological basis for potential effect of childhood vaccines in disease expression	(10)	Salman S, Salem M	Egypt	Hypothesis
	(14)	O'Neill LA, Netea MG	Ireland	Hypothesis
	(15)	Sabir DK, Sidiq KR, Ali SM	Iraq	Genomic data analysis
	(16)	Abdulamir AS, Hafidh RR	Iraq	Hypothesis
The bacillus-calmette-guerin (BCG) vaccine	(17)	Miller A, Reandelar MJ, Fasciglione K, Roumenova V, Li Y, Otazu GH	Global	Regression analysis
	(18)	Shet A, Ray D, Malavige N, Santosham M, Bar-Zeev N	United States of America	Regression analysis
	(19)	Escobar LE, Molina-Cruz A, & Barillas-Mury C	Global	Regression analysis
	(20)	Escobar LE, Molina-Cruz A, & Barillas-Mury C	Global (2)	Regression analysis
	(21)	Paredes JA, Garduño V, Torres J	Mexco	Regression analysis
	(22)	Hamiel U, Kozer E, Youngster I	Israel	Regression analysis
The measles, mumps, and rubella (MMR) vaccine	(23)	Saad M & Elsalamony R	Egypt	Hypothesis
	(24)	Franklin R, Young A, Neumann B, Fernandez R, Joannides A, Reyahi A, et al.	United Kingdom	Genomic data analysis
	(25)	Fidel PL, Noverr MC	United States of America	Hypothesis
The Hepatitis-A (HEP-A) vaccine	(26)	Sarialioglu F, Apak FBB, Haberal M	Turkey	Hypothesis

### The Immunological Basis for Potential Effect of Childhood Vaccines in Disease Expression

In many countries, children are routinely vaccinated against a number of bacterial and viral diseases. Vaccines may have non-specific physiologic effects when they alter the immune response to unrelated organisms, called heterologous immunity. The non-specific effects of vaccines are usually more pronounced in girls and appear to be maximal in the first 6 months of life (11)—when passed maternal immunity is further supplemented by newly introduced vaccines, starting at 2 months. There are several theories as to why heterologous immunity may occur.

Salman and Salem suggest that cross-immunogenicity of childhood vaccines for multiple viruses could potentially be a reason for the relatively milder infection and severity of COVID-19 among children (10). Most routine viral vaccines are either inactivated or killed viruses that stimulate T Helper 1 cells (CD4+) to secrete many different types of cytokines as interferon gamma, interleukin-2 (IL-2), and IL-12, improving the cytotoxicity of natural killer cells to recognize and destroy cells infected with new cross-reactive viruses. For example, warts that are caused by human papilloma virus (HPV) could be ameliorated using intralesional MMR vaccine (10).

Furthermore, neutralizing antibodies produced against the foregoing vaccine-preventable microbes might cross-react with the antigenic epitopes of the spike (S) and nucleocapsid (N) proteins and prevent COVID-19 in children (15). An investigation of this hypothesis, using the BLAST search tool, showed no significant sequence similarity between these proteins and those in the childhood vaccine-preventable microbes, inferring that memory T-cells, rather than vaccine neutralizing antibodies, may be involved in the protection of children against COVID-19 owing to them having a larger number of naive T-cells that can be programmed to protect them against the disease (27).

Potentially, the low immunity in children that doesn't exaggerate the immune response against the virus as in the case of adults, could explain the lesser severity of SARS-CoV-2 in this age group. Children have less adults-like memory cells specific to other circulating coronaviruses and therefore, are less capable to mount a devastating and vigorous cell-mediated attack on alveoli and interstitial tissue of the lung upon new infection (16).

### The Bacille Calmette–Guérin (BCG) Vaccine

The Bacille Calmette-Guérin (BCG) vaccine is given in infancy for prevention of severe forms of tuberculosis and has the widest use, and a strongest safety profile among all childhood vaccines (28). Epidemiological and randomized trial evidence suggest a protective effect of BCG on infant mortality via non-specific heterologous protection against other infections possibly through innate immune epigenetic mechanisms (29).

O'Neil and Netea suggest that induction of trained immunity by BCG vaccine could provide protection against COVID-19, and the use of oral polio vaccine and new recombinant BCG-based vaccine VPM1002 may be some of the approaches to induce resistance to SARS-CoV-2 (14). The authors hypothesize that induction of trained immunity is at least partly the mechanism through which BCG vaccination induces its beneficial effects and might protect against SARS-CoV-2.

A retrospective study compared countries that do not have BCG vaccination policies (Italy, USA, Lebanon, the Netherlands, and Belgium), to countries that have such policies (17). The results showed that while middle-high and high-income countries with current universal BCG policies had 0.78 COVID-19 deaths per million, those without such policies had 16.39 COVID-19 deaths per million people—and the difference was statistically significant. Further analysis of 28 countries found a positive significant correlation (*p* = 0.02) between the year of the universal vaccination policy and mortality rate—suggesting that if the policy to vaccinate was adopted at an earlier year, more of the elderly population in these countries would have been vaccinated, thus potentially offering them more protection. In countries, such as Italy, where BCG vaccine was never given, the mortality rate was significantly higher compared to Japan where BCG vaccination has been implemented since 1947. In countries, such as Iran, with BCG vaccination starting in 1984, mortality was higher since today's elderly population did not receive the vaccination.

In order to mitigate the bias centered around the differential epidemic time curves experienced by different countries, Shet et al., calculated days from the 100th COVID-19-positive case to align countries on a more comparable time curve (18). A log-linear regression model was built with crude COVID-19-attributable mortality data per 1 million population for each country as outcome, BCG vaccine inclusion in the national immunization schedule as exposure, and adjusted for the effects of: country-specific GDP per capita, the percentage of population 65 years and above, and the relative position of each country on the epidemic timeline. COVID-19-attributable mortality among BCG-using countries was 5.8 times lower (*P* = 0.006) than in non-BCG-using countries. Sensitivity analysis run excluding China as the majority case contributor from the model resulted in no appreciable change in the protective effect of BCG.

Escobar et al., in a study that carefully controlled for confounding variables found that there was an inverse correlation between countries/locations with a stronger BCG vaccination policy and COVID-19 related mortality (19). COVID-19 mortality rates in New York, Illinois, Alabama and Florida—states without BCG-vaccination policies in the US, were significantly higher than locations with BCG-vaccine policies, namely Pernambuco, Rio de Janeiro, and Sao Paulo in Brazil, or Mexico State and Mexico City in Mexico.

In a more recent study, the same authors demonstrate a strong correlation between the BCG index and COVID-19 mortality in different socially similar European countries (*r*^2^ = 0.88; *P* = 8 × 10–7), indicating that every 10% increase in the BCG index was associated with a 10.4% reduction in COVID-19 mortality (20).

However, evidence suggesting a protective effect of the BCG vaccine was not found to be universally consistent and only demonstrated association not causality. There indeed are a myriad of factors apart from the effect of a childhood vaccine that could impact the findings of association, and such caution in interpretation would be recommended—especially this early in our understanding of the COVID-19 disease.

Paredes et al., showed that when confounders such as under-reporting, SARS-CoV-2 capability testing and differing lockdown measures were considered, the differential impact of BCG vaccination on COVID-19 related mortality rate was not significant (21). Among high-income countries, the mean number of deaths per 1 million population for countries with no universal BCG vaccination (223.2 ± 166.1) was not statistically significant from countries with current or previous BCG vaccination programs (55 ± 82.5; *P* = 0.85). No statistically significant difference was noted in mean number of deaths at the 1,000th case in these three groups either.

Hamiel et al., compared infection rates and proportions with severe COVID-19 disease in 2 cohorts: individuals born during 3 years before and 3 years after cessation of the universal BCG vaccine program in Israel (22). There was no statistically significant difference in the proportion of positive reverse transcriptase-polymerase chain reaction tests for SARS-CoV-2 in the BCG vaccinated group compared to the unvaccinated group (11.7 vs. 10.4%, *p* = 0.09). There also was no statistically significant difference in positivity rates per 100,000 (121 vs. 100, *p* = 0.15).

### The Measles, Mumps, and Rubella (MMR) Vaccine

Saad at al., suggested two potential mechanisms for higher COVID-19 cases per population ratio and higher death rate in Italy (no MMR vaccine) compared to China: (1) by generating bystander immunity the measles vaccine increases ability of immune system to combat non-measles pathogens, including coronaviruses, and (2) due to shared structural similarities between measles and coronavirus the cross-reactivity and immunity between the measles vaccine and coronavirus leads to partial protection against COVID-19 (23).

Franklin et al., identified that the macro domains of SARS-CoV-2 and rubella virus and the MMR vaccine, share 29% amino acid sequence identity (24). This finding suggests the viruses possess the same protein fold. Patients with high illness severity had high levels of rubella IgG (161.9 + 147.6 IU/ml) compared to patients with a moderate severity of disease (74.5 + 57.7 IU/ml). The authors suggest the MMR vaccine could result in potentially reduced severe outcomes with COVID-19.

In their commentary, Fidel and Noverr support the use of live attenuated MMR vaccine as a preventive measure against the pathological inflammation and sepsis associated with COVID-19 infection (25). While they emphasize the strictly preventive nature of the suggestion, the basis of such suggestion is the induction of non-specific effects by live attenuated vaccines that represent “trained innate immunity” delivered by leukocyte precursors in the bone marrow more effectively functioning against broader infectious attacks. On the basis of data from prior BCG trials in infants, the vaccine-induced trained innate cells are expected to remain in the circulation for roughly 1 year, which should see people through the most severe waves of COVID-19 infection.

### The Hepatitis-A (HEP-A) Vaccine

Sarialioglu et al., reported on the differences in the rate in which COVID-19 had affected some countries such as China, US, Italy, Spain, France, England, the Netherlands, and Belgium more severely than some others such as India, Pakistan, countries of the African continent, and South America which had lower rates of infection and mortality at the time of their study (26). The authors hypothesize that routine vaccination for hepatitis A virus (HAV) causing high seroprevalence among populations in countries in the low COVID-19 prevalence group, while it is rather low in the industrialized countries.

In addition, the authors point to the COVID-19 experience in the Diamond Princess cruise ship, which after arriving in Yokohama, Japan on February 3rd 2020, was placed under quarantine for the disease based on another passenger who had disembarked in Hong Kong a couple of days earlier and has tested positive for the virus (30). A report (31) showed that by February 20th over 18% of the 700 infected among the 3,700 people showed no symptoms. The low frequency of symptomatic disease on the ship, may be explained by stimulated immunity before passengers started the cruise trip when HEP-A vaccine was recommended for international travel in areas with high HAV endemicity. However, no publicly available information on the HEP-A vaccination status of the passengers were found.

While there does not seem to be any objective evidence to support this yet, the authors further contemplate that the severity of COVID-19 and vulnerability of very young children, particularly infants <1 year of age, may be attributed to the eventual decrease of maternal anti-HAV antibodies toward age 1 year—as HEP-A vaccine is not administered until after 1 year of age.

The authors conclude that immune response caused by the hepatitis A vaccine may be protective against COVID-19 infection by a possible adaptive immune cross-reaction. Patients with asymptomatic COVID-19 disease could indirectly indicate those with protection from HAV seropositivity. The HEP-A vaccine may help to keep the COVID-19 infection at mucosal colonization levels and prevent lower respiratory tract involvement and fatality (26).

## Discussion

At the time of this writing, the pandemic of COVID-19 continues to be a global public health emergency, claiming the lives of hundreds, and infecting millions all over the world. While the trend thus far shows a relatively less severe morbidity and mortality profile of the disease among children, the reason behind such a trend is not yet well-understood. While several theories for such welcome relief have been proposed, we present available insights and hypotheses on the potential link between childhood vaccination and the less severe expression of COVID-19 in this early narrative review.

Although it is relatively early in the process of the scientific community's gaining full understanding of the SARS-CoV-2 virus and characterization of its infection, known virus-prevention strategies from past pandemics that could lead to potential attenuation of the currently ongoing disaster, are of high interest. Public health emergencies, by their nature, often do not have the luxury of time needed for well-researched remedies, and that is why hypotheses and theories are relevant—even if with the possibility of bridging current patients and populations to the time when treatment and vaccination for COVID-19 are available.

Our narrative review finds that there indeed is a potential scientifically-based possibility of heterologous immunity from common childhood vaccines to be imparting a protective effect on COVID-19 infections in children. While not unequivocal, population-level differences found in several studies in the rate of infection and severity of expression of COVID-19 between countries with and without certain common childhood vaccination policies suggest the need for deeper and more well-structured investigation, in the minimum. Although prevalence of some target diseases and organisms may have been eradicated in certain parts of the world, the reinstitution of relatively inexpensive vaccines for those diseases into the currently recommended childhood vaccination regimen may merit careful re-evaluation. Our review found suggestions from the medical community of such promise in at least three of the most common vaccines given to children—BCG, MMR, and HEP-A.

However, it is indeed not recommended that such practices be instituted without establishing a reasonable scientific evidence to validate some of these hypotheses—especially when children are involved. Pragmatic randomized controlled trials designed to time- and cost-efficiently test feasible primary endpoints of cross-immunogenicity with existing childhood vaccines should be initiated alongside focused global efforts to develop effective treatment for COVID-19, and a safe and effective SARS-CoV-2 vaccine. At least, rapid testing and eventual use of promising, non-COVID-19 vaccines could be explored for help with avoiding large patient casualties in the meantime, until adequate treatment and vaccines are developed.

Certainly, routine pediatric vaccination for other conditions needs to be maintained even in the face of parental fear of potential exposure to COVID-19 during well child visits. Parents need to be reminded of the increased risks for outbreaks of vaccine-preventable diseases that children and their communities may face upon lifting of social distancing guidelines—unless children are vaccinated appropriately.

## Conclusion

Based on our review, it may be concluded that although controlled clinical trials may be time and resource intensive, those may be justificable in investigating further and confirming the value of at least three childhood vaccines: BCG, MMR, and HEP-A as possible explanations for lower incidence of COVID-19, and less severe expression of the disease in children. Currently hypothesized explanations for an evidently less severe impact of COVID-19 on children globally includes the protective cross-immunity provided by other common childhood vaccines. There is a strong basis to hypothesize that immune response to the SARS-CoV-2 virus in children is different than in adults, resulting in differences in the levels of severity of symptoms and outcomes of the disease in different age groups.

## Author Contributions

Upon initial suggestion of the topic by the BB-S, JK-H, DR, and SR participated equally in generating the research question, conducting library search, and writing the manuscript and selected articles were divided equally for review and data entering into the common spread sheet. All authors participated in revising and editing the manuscript.

## Conflict of Interest

The authors declare that the research was conducted in the absence of any commercial or financial relationships that could be construed as a potential conflict of interest.
